# Ventricular Fibrillation in an Afebrile COVID-19 Patient Presenting With Transient Type-I Brugada Pattern

**DOI:** 10.7759/cureus.38220

**Published:** 2023-04-27

**Authors:** Judah A Kreinbrook, Annalia Foster, Luis Paulino, Fabio Leonelli

**Affiliations:** 1 School of Medicine, Duke University, Durham, USA; 2 Dr. Kiran C. Patel College of Osteopathic Medicine, Nova Southeastern University, Clearwater, USA; 3 Internal Medicine, University of South Florida Morsani College of Medicine, Tampa, USA; 4 Cardiology, James A. Haley Veterans' Hospital, Tampa, USA

**Keywords:** afebrile, ventricular fibrillation, electrocardiography, brugada phenocopy, covid-19, brugada syndrome

## Abstract

COVID-19 has been associated with an increased risk of both atrial and ventricular arrhythmias. Brugada syndrome (BrS), an inherited sodium channelopathy presenting with a characteristic ECG morphology, confers a baseline risk of ventricular arrhythmias such as ventricular fibrillation (VF), especially during febrile illnesses. However, mimics of BrS, termed Brugada phenocopies (BrP), have been noted in association with fever, electrolyte abnormalities, and toxidromes outside of viral illness. Such presentations manifest the same ECG pattern, the type-I Brugada pattern (type-I BP). Thus, the acute stage of an illness such as COVID-19, when accompanied by a first-time presentation of type-I BP, may not result in a certain diagnosis of BrS versus BrP. Thus, expert recommendations are to anticipate arrhythmia regardless of the presumed diagnosis. Here we demonstrate the importance of these guidelines and a novel report of VF in the setting of a transient type-I BP in afebrile COVID-19. We discuss the potential factors which may have triggered VF, the presentation of isolated "coved" ST elevation in V1, and the difficulty of BrS versus BrP diagnosis in acute illness. In summary, a SARS-CoV-2 positive 65-year-old male without significant cardiac history for BrS presented with type-I BP after two days of shortness of breath. Hypoxemia, hyperkalemia, hyperglycemia, elevated inflammatory markers, and acute kidney injury were present. After treatment, his ECG normalized; however, aborted VF occurred days later while afebrile and normokalemic. Follow-up ECG again revealed a type-I BP, which also became more apparent during an episode of bradycardia, a classic finding in BrS. This case suggests that there is room for larger studies to determine the prevalence and outcomes when type-I BP presents in acute COVID-19. When possible, genetic data should be obtained to confirm BrS, a notable limitation in our case. Regardless, it corroborates guideline-directed clinical management, with heightened vigilance for arrhythmia in such patients until full recovery.

## Introduction

COVID-19, the disease caused by the RNA virus SARS-CoV-2, is associated with arrhythmias in hospitalized patients [1]. Infection-associated myocarditis, pericarditis, myocardial injury, electrolyte abnormalities, febrile conditions, and inflammatory response, all common in hospitalized patients, are linked to this increase in arrhythmogenic potential [2]. When this occurs alongside inherited channelopathies with an underlying risk of arrhythmia, such as Brugada syndrome (BrS), caution is warranted based on the underlying pathophysiology and expert recommendations [2-3]. However, BrS is difficult to diagnose in acute illness, especially as it can present on ECG without cardiac symptoms. The classic ECG presentation, the type-I Brugada pattern (type-1 BP), is defined as ≥2 mm of "coved" ST-segment elevation (STE) in one or more high precordial leads (i.e., V1, V2, and/or V3) with negative symmetric T-wave inversion, a finding which may also be present in Brugada phenocopies (BrP) [4]. The latter are acquired, temporary, and usually benign sodium channelopathies that mimic BrS [4-5]. As highlighted in a recent narrative review, numerous case reports and series have demonstrated the occurrence of type-I BP in COVID-19; however, it is unclear if these cases were unmasked BrS or BrP in the setting of COVID-19. Such an interpretation is limited by heterogeneous presentations, diverse management strategies, lack of genetic testing, and a usually benign course of COVID-19. Currently, retrospective research on this presentation, outside of such case reports, series, and expert opinion, is lacking. 

Expert opinion, while mostly reliant on data obtained outside of COVID-19, suggests management, regardless of presumed BrS versus BrP diagnosis, should focus on minimizing arrhythmogenic triggers such as fever [6-7]. However, reduction of fever alone, unlike in other viral syndromes, may not be sufficient. This is due to reports of afebrile cases, suggesting a role for the inflammatory response itself as an arrhythmogenic trigger [2,8-9]. Additionally, severe COVID-19 often involves multiple organs, disrupting normal electrolyte levels, often secondary to acute kidney injury (AKI). This indirectly elevates arrhythmogenic potential [2]. 

Along these lines, we report an occurrence of ventricular fibrillation (VF) in a patient who presented with a type-I BP in severe COVID-19. In this case, an afebrile COVID-19 patient with no known personal or family history of BrS presented with coved ST elevation in lead V1 which met the criteria for type-I BP. Additionally, AKI was present alongside associated hyperkalemia, hyponatremia, and hyperglycemia, complicating diagnosis and management. Following the correction of these abnormalities, the patient's ECG normalized, with the clinical team focusing on respiratory management. His ECG was deemed to represent "non-specific ST-T wave changes"; however, the patient experienced VF days later while afebrile, normokalemic, normonatremic, and in normal sinus rhythm.

## Case presentation

**Figure 1:**
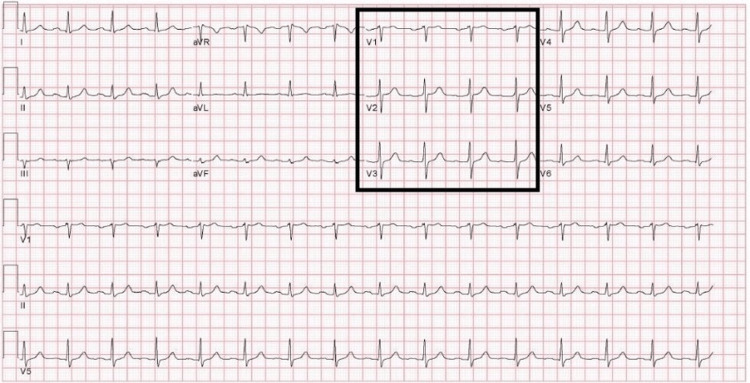
Historical ECG taken 12 years prior Note no STE in V1-V3 and upright T-waves. STE - ST-segment elevation

**Figure 2:**
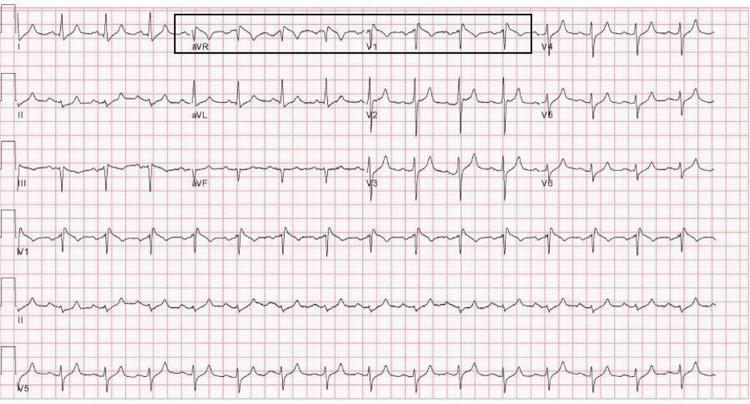
ECG taken upon presentation to ED Note STE in V1 and aVR with coved-type morphology, representing a type-1 BP. Severe hyperkalemia was present (7.5 mmol/L). STE - ST-segment elevation; type-1 BP - type-I Brugada pattern

A 65-year-old male with a history of obesity, hypertension, dyslipidemia, liver cirrhosis, treated hepatocellular carcinoma, and treated hepatitis-C infection presented to the emergency department (ED) after two days of progressive shortness of breath, malaise, and a positive SARS-CoV-2 polymerase chain reaction (PCR) test. Vaccination with two doses of BNT162b2 was completed six months prior. He reported no cardiac history, including a negative family history of sudden cardiac death and previously normal ECGs (Figure 1). On presentation, his initial ECG revealed a type-I BP with ST-segment elevation (STE) present in V1 and aVR (Figure 2). Although the automated interpretation read "type-I Brugada pattern", the clinical team focused on respiratory management and admitted to ICU, deeming the pattern to represent non-specific ST-T changes.

Upon arrival, he was afebrile and hypoxic with oxygen saturation (SpO_2_) of 83% on room air, improving to 92% on 6 lpm of molecular oxygen (O_2_) via nasal cannula. Laboratory results revealed serum sodium of 120 mmol/L (130 mmol/L, corrected using blood glucose for accounting possible pseudo-hyponatremia), potassium of 7.5 mmol/L, lactic acid of 2.1 mmol/L, pH of 7.41, partial pressure of oxygen (pO_2_) of 70 mmHg, partial pressure of carbon dioxide (pCO_2_) of 29 mmHg, chloride of 94 mmol/L, blood urea nitrogen (BUN) of 103 mg/dl, creatinine of 4.6 mg/dL, elevated C-reactive protein (CRP) of 5.8 mg/dL, ferritin of 1551 ng/mL, D-dimer of 939 ng/mL, and blood glucose of 753 mg/dL. High-sensitivity cardiac troponin (hs-cTn) and b-type natriuretic peptide (BNP) were within normal limits. Diagnoses of acute respiratory distress syndrome (ARDS), acute kidney injury (AKI), non-anion gap metabolic acidosis (NAGMA), and hyperosmolar non-ketotic syndrome (HHNS) were made as serum beta-hydroxybutyrate was within normal limits at 1.3 mg/dL (reference value: 0-2.8). No personal or family history of BrS was reported.

**Figure 3:**
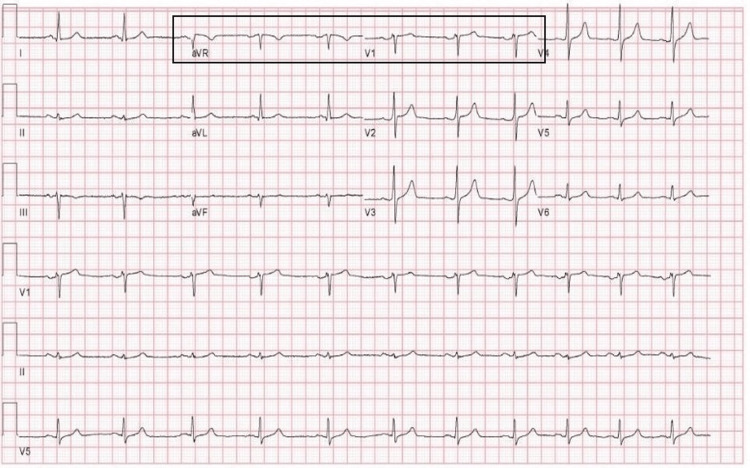
ECG taken on day 1 of admission Note the normalized ST-segments in V1 and aVR. Mild hyperkalemia was present (5.7 mmol/L) at this time.

Heated-high flow nasal cannula (HF-NC) was initiated alongside insulin, normal saline, calcium gluconate, acetaminophen, and albuterol. Serum potassium reached a low of 4.5 mmol/L the following day alongside serum sodium of 136 mmol/L, and serum glucose remained <200 mg/dL. Hypoxemia improved slightly to 77 mmHg. ECG obtained the following day normalized (Figure 3). Throughout hospitalization, he remained afebrile, normonatremic, and relatively normokalemic (4.5 to 6.2 mmol/L), with frequent episodes of hyperglycemia controlled via insulin infusion. His creatinine improved to 2.3 mg/dL with a BUN of 94 mg/dL, indicating improvement of renal function with treatment. Etiology was presumed to be pre-renal AKI with consideration of acute tubular necrosis on the differential. Over the coming days, his renal function remained stable; however, his respiratory status progressively declined, requiring increased O_2_ flow/fraction of inspired oxygen (FiO_2_; 60L/90%). A PO_2_ of 60 mmHg was noted on the arterial blood gas taken on the morning of hospitalization day 8. Blood glucose was 157 mg/dL, potassium was 5.3 mmol/L, and sodium was 134 mmol/L. 

**Figure 4:**
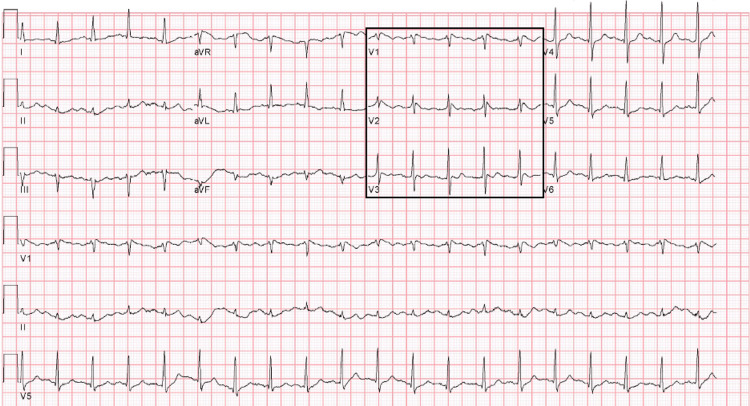
ECG taken 30 minutes after the episode of ventricular fibrillation Note the reappearance of type-I BP as evidenced by STE in V1 and V2 with coved-type morphology. Mild hyperkalemia was present (5.7 mmol/L) alongside severe acidosis (pH 7.01). STE - ST-segment elevation; type-1 BP - type-I Brugada pattern

**Figure 5:**
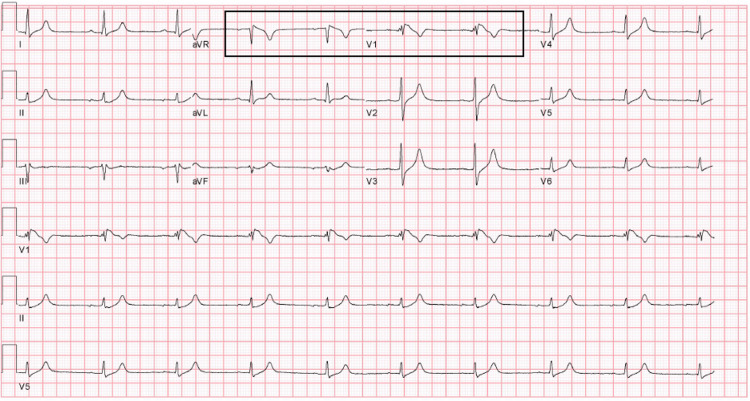
ECG taken four hours after the episode of ventricular fibrillation Note type-I BP as evidenced by STE in V1 and aVR with a widened coved-type morphology compared to Figures 2 and 4. Moderate hyperkalemia was present (6.9 mmol/L) alongside severe acidosis (pH 7.01). STE - ST-segment elevation; type-1 BP - type-I Brugada pattern

Five hours later, an infusion of dexmedetomidine was started to relieve anxiety, with no bradycardia noted on telemetry or documented by the team. Approximately forty-five minutes after starting the infusion, he demonstrated ventricular fibrillation while in normal sinus rhythm and was afebrile during the morning assessment (98.2° Fahrenheit). Upon successful return of spontaneous circulation (ROSC) after delivery of single defibrillation to abort VF, a type-I BP was again noted on ECGs taken 30 minutes and four hours after VF episode (Figures 4-5). His first post-VF ECG (Figure 4) occurred under mild hyperkalemia (5.7 mmol/L), and the second (Figure 5) under more moderate hyperkalemia (6.9 mmol/L). While previously normal, pH was acidotic post-VF (pH 7.01). The bedside echocardiogram was normal, ruling out structural disease, and a repeat hs-cTn was within normal limits.

He was intubated post-resuscitation due to hemoptysis. During this time, he underwent an episode of bradycardia, resulting in the accentuation of the type-1 BP (Figure 5). Ongoing hypotension was managed with fluid resuscitation and vasopressors, with a steady decline of mean arterial pressures despite therapies. Care was withdrawn the following day.

## Discussion

The type-I Brugada ECG pattern is a classic presentation of the heritable BrS and the acquired BrP. Evidence demonstrates that the ECG morphology in both cases is not discernible by expert electrocardiologists in blinded conditions [10]. In our case, there may be debate as to its qualification as a type-I BP due to it only lying in one lead, V1. Recent consensus confirms that one or more leads (V1-V3) of ST elevation are sufficient [11]. Notably, this criterion was incorporated into the guidelines after the classic multi-lead criterion was overturned [12].

Fever has been a well-described precipitant of ventricular fibrillation (VF), with demonstrated sodium channel inactivation [13]. Previous case reports of type-I BP in COVID-19 frequently note fever alongside elevated inflammatory markers, a typical severe COVID-19 presentation. However, in one case, no fever, inflammatory marker elevation, or AKI was present, with the authors suggesting a direct role for SARS-Cov-2 in its interaction with the cardiac sodium channel [14]. At a minimum, fever alone cannot explain this case, and to our knowledge, it is the only published report of type-1 BP in COVID-19 that lacked fever, elevated inflammatory markers (e.g., CRP), and abnormal serum electrolytes. Thus, the direct involvement of SARS-Cov-2 with the cardiac sodium channel should still be confirmed [9,14].

Our case demonstrates what we believe is the first reported occurrence of a transient type-I BP ECG pattern first resolving before reappearing after an episode of aborted VF in COVID-19. This is atypical when compared to published reports which generally show few arrhythmias and a successful hospital discharge with a normal ECG in more than 80% of cases [2,9]. Upon our brief search of the literature, we identified two other cases of ventricular arrhythmia in the setting of type-I BP and COVID-19. One was suspected to have BrS prior to acquiring COVID-19, with an implantable defibrillator terminating VF. He was later confirmed to have BrS via genetic testing, which revealed a heterozygous mutation in SCNA5. Such testing was obtained because of an unclear family history of syncope and sudden cardiac death. His son suffered a single episode of syncope presumed to be vasovagal, his father died at 75 with an extensive cardiac history, and his grandfather died suddenly in his 50s [15]. In the remaining case, no family history or previous suspicion was present. It was posited that dexmedetomidine-induced bradycardia under febrile conditions resulted in polymorphic ventricular tachycardia (PMVT) [16]. Regarding dexmedetomidine, it is not recommended in cases of suspected BrS or BrP [7]. While dexmedetomidine was also used in our case prior to VF, we noted no bradycardia, suggesting that its effects on vagal tone may have been negligible. Nevertheless, this cannot definitely be ruled out, and dexmedetomidine should be avoided [7].

Such cautious management is warranted as COVID-19 infection alone elevates arrhythmic risk [3,17]. It has been suggested that this occurs due to direct effects such as myocarditis, abnormal host immune response, and hypoxic lung injury due to viral infiltration. These may be combined with indirect effects such as myocardial ischemia, medication side effects, intravascular volume derangements, and myocardial strain due to pulmonary hypertension [2]. As noted in our case, COVID-19 often results in AKI, with the requisite electrolyte abnormalities also increasing arrhythmogenic potential [2,17]. While correction of these electrolytes was associated with type-I BP resolution in our case, the episode of VF and subsequent follow-up ECGs suggest that sodium channel dysfunction, whether from unmasked BrS or acquired BrP, may have remained. Although structural heart disease or undiagnosed myocarditis were potential contributors, these were ruled out via negative high-sensitivity troponin and a normal trans-thoracic echocardiogram post-ROSC.

Hyperkalemia, hyponatremia, and acidosis were present early in this case; nevertheless, we can be reasonably certain that the contribution of hyperkalemia to VF was minimal as the same potassium level of 5.7 mmol/L was seen in the normalized ECG (Figure 3) and the ECG showing type-I BP after resuscitation (Figure 4). Serum sodium levels were also corrected prior to the normalized ECG and remained within normal limits after resuscitation. Though acidosis was seen post-resuscitation, pH was normal hours prior, suggesting this was not present at the time of VF.

Other arrhythmogenic factors likely contributed to this event. Hypoxemia and elevated cytokines, both present throughout hospitalization, have well-documented arrhythmogenic mechanisms and have been noted in case reports [1,17]. This illustrates the importance of considering all patients with severe COVID-19 to be in a potentially arrhythmogenic state. With the addition of a potential heritable or acquired channelopathy, caution should be exercised [1].

Regarding the final diagnosis of an unmasked BrS versus acquired BrP, two events appear relevant: the resolution of the type-I BP and its reappearance after resuscitation [4,5]. Its original resolution may have either been related to the initial correction of hyperkalemia, hyponatremia, hyperglycemia, or simply part of the natural history of an unmasked BrS. Likewise, its reappearance was under acidotic conditions. Thus, neither aid in the diagnosis of BrS versus BrP. Such a diagnosis must be made after acute illness abates, a condition requiring patient survival [6].

Nevertheless, the accentuation of the type-I BP during bradycardia is a finding classically associated with BrS [18]. Such an event suggests that sodium channel inactivation was present, regardless of the level of heritability (i.e., BrS versus BrP). While analyses of the often implicated SCNA5 gene may have served to confirm the diagnosis of BrS, we were unable to obtain this sample. Such tests also lack sufficient sensitivity to rule out an underlying BrS, with estimates of approximately 30% [6]. A sodium channel blocker challenge may have also aided in diagnosis; however, this should not be performed in acute illness [5,6].

Regardless of the specific diagnosis, our case suggests that diagnosis may not be feasible, adding context to the current expert recommendations on this presumably rare presentation [6,7]. When presented with new-onset type-I BP in COVID-19, daily 12-lead ECGs, diligent correction of all potential arrhythmogenic factors, telemetry, and application of defibrillation pads should be considered. Additionally, our case corroborates previous work, which sought to collect the existing case reports and series regarding a potential association between type-I BP and arrhythmias in COVID-19 [2,9]. Further large multi-center retrospective studies are needed to determine its prevalence, outcomes, and prognosis, guiding management.

## Conclusions

Severe COVID-19 can result in numerous direct and indirect effects on arrhythmogenic potential, including but not limited to hypoxemia, electrolyte abnormalities, and elevated inflammatory markers. Our case is potentially the first to demonstrate these in association with a transient type-I BP which reappeared after an episode of ventricular fibrillation. While cases such as this may have a difficult diagnosis (i.e., unmasked BrS versus acquired BrP), this may be difficult without patient survival and/or confirmation via genetic testing, a test which lacks sufficient sensitivity. Nevertheless, this case adds to the weight of the relevant expert recommendations, suggesting that acute management should assume elevated arrhythmogenic potential and anticipate ventricular arrhythmias in these patients regardless of diagnosis. While further reports of such cases will be important, we suggest there is room for larger retrospective studies to determine its prevalence in COVID-19 and to guide management.
